# Effects of a *saccharomyces* yeast postbiotic on growth performance and carcass traits of pigs fed diets with divergent protein content

**DOI:** 10.1093/tas/txag013

**Published:** 2026-02-12

**Authors:** X Li, D A Alambarrio, S S Zedonek, M A Vaughn, C B Paulk, J M Gonzalez

**Affiliations:** Department of Animal and Diary Science, University of Georgia, Athens, GA 30602, United States; Department of Animal and Diary Science, University of Georgia, Athens, GA 30602, United States; Department of Animal and Diary Science, University of Georgia, Athens, GA 30602, United States; Puretein Bioscience, LLC, Minneapolis, MN 55416, United States; Department of Grain Science and Industry, Manhattan, KS 66506, United States; Department of Animal and Diary Science, University of Georgia, Athens, GA 30602, United States

**Keywords:** elevated-protein, feed performance, porcine, *Saccharomyces* yeast postbiotic, yield

## Abstract

The objective of this study was to determine effects of a *Saccharomyces* yeast postbiotic (**SYP)** on pig growth performance and carcass traits when added to diets containing elevated- and commercial-protein contents during growing and finishing phases. Pigs (*N = *51 barrows and 57 gilts; initial BW 28.3 ± 3.5 kg) were weighed, stratified by weight within sex, and within each 18-pig strata pigs were randomly assigned to a pen (*N = *18; *N = *6 pigs/pen). Pens were randomly assigned to one of two dietary treatments (**TRT**) consisting of 0- (**SYP-**) or 100-ppm SYP (**SYP+**) mixed in a four-phase dietary regimen (phase 1, d 0 to 21; phase 2, d 22 to 42; phase 3, d 43 to 63; phase 4, d 64 to 84). Feed consumption and body weight (**BW**) were collected weekly to calculate average daily gain (**ADG**) and average daily feed intake (**ADFI**). Pigs were harvested over three harvest blocks where the heaviest male and female from each pen were harvested and carcass data were collected 24-h postmortem. During all four phases, there were no TRT effects on BW, ADG, and ADFI (*P >* 0.12), except SYP + pigs had less (*P = *0.04) phase-4 ADFI. There was no TRT effect (*P = *0.86) on phase-2 G: F, but SYP + pigs had greater phase-1 and -4 G: F and smaller phase-3 G: F (*P <* 0.04). Carcasses from SYP + pigs tended to have greater (*P = *0.09) HCW and had greater (*P <* 0.01) dressing percent than SYP- carcasses. There were no TRT treatment effects for 10^th^ or last rib fat thickness (*P >* 0.13), but SYP + carcasses tended to have less (*P = *0.07) first rib fat thickness. There were no TRT effects on loin eye area, objective- and subjective-color scores (*P >* 0.24). Carcasses from SYP + had smaller NPB marbling and Japanese marbling color scores than SYP- carcasses (*P <* 0.02). This SYP mainly enhanced feed conversion efficiency and lean-derived carcass traits when added to an elevated-protein diet.

## Teaser

This study demonstrated a *Saccharomyces* yeast postbiotic supplement enhanced pig growth efficiency and possibly protected against intestinal disease. These findings provide producers with information on dietary protein content and disease effects on the SYP response.

## Introduction

Over the past 50 years, global population has grown rapidly, leading to an increase in meat demand (Henchion et al. 2014). Pork is a major animal-derived protein source, accounting for 30% of global meat consumption (Chernukha et al. 2023; Dehelean et al. 2023; Fan et al. 2023). This surge in pork consumption necessitated a parallel increase in production, a process that hinges not only on efficient pig breeding but also on maximizing feed efficiency (Agostini et al. 2014). Losinger (1998) reported feed was the ­foundation upon which pig health, growth rate, and overall productivity were improved. Therefore, improving pork production efficiency through dietary manipulation has consistently attracted scientists’ and producers’ interest.

Over millennia, humans have utilized *Saccharomyces* cerevisiae (**SCE**), a single-celled fungal microorganism, in baking, brewing, and wine making. In early livestock research, Newbold et al. (1996) reported this microorganism was an excellent ruminant feed fiber source and Elghandour et al. (2020) found SCE promoted pigs’ feed intake, increased growth performance, and enhanced intestinal development and function. celluTEIN is a *Saccharomyces* yeast postbiotic (**SYP**) with sub-nutritional levels of arginine and leucine that assist the unique yeast to target the mechanistic target of rapamycin (**mTOR**) pathway which stimulates in vitro muscle protein deposition (Vaughn and Gonzalez 2022). In field trials, SYP included in traditional multiple-phase swine diets improved nursery growth performance measures (Kim and Duarte 2024) and from weaning through finishing (Vaughn et al. 2023). When feed supplements drastically alter muscle protein synthesis, nutritionists may need to alter dietary nutrient requirements. For example, to accommodate enhanced protein accumulation associated with ractopamine-HCl supplementation, Jacela et al. (2009) reported in addition to the product label stating dietary protein should be increased to 16% and standardized ileal digestible-lysine should also be increased by 0.3% (Manu and Baidoo 2020). To date, elevated protein and amino acid inclusion levels of SYP supplemented diets remain unexplored. Therefore, this study aimed to evaluate SYP effects on pig feed performance and carcass characteristics when included in elevated- or commercial-protein growing and finishing diets.

## Materials and methods

The University of Georgia Institutional Animal Care and Use Committee approved the protocol used in this experiment (A2023 01–019-Y2-A0).

### Experiment #1

#### Live animal management and diets

Pigs (51 barrows and 57 gilts; Camborough × PIC 337; Pig Improvement Company, Hendersonville, TN, USA; initial BW 28.3 ± 3.5 kg) were transported from the University of Georgia Swine Unit (Athens, GA) to the University of Georgia Large Animal Research Unit (**LARU**; Athens, GA). Upon arrival, pigs were weighed, stratified by weight within sex, and within each 18 pig strata, randomly assigned to a pen (*N = *18; 6 pigs/pen). Pigs were housed in an environmentally controlled room and pens were 7.5 m^2^ with solid-concrete slatted floors, a 2-hole dry feeder (Farmweld, Teatopolis, IL) and a nipple waterer enabling ad libitum access to feed and water. Pens were randomly assigned two dietary treatments consisting of conventional swine finishing diet containing 0 (**SYP-**; *n = *9) or 100 ppm (**SYP+**; *n = *9; celluTEIN; Puretein Bioscience LLC, Minneapolis, MN). Diets were prepared at Godfrey’s Feed (Madison, GA) and fed in four-phases (phase 1, d 0 to 21; phase 2, d 22 to 42; phase 3, d 43 to 63; phase 4, d 64 to 84; Table 1). All pigs were fed phase 1 SYP- feed during the first 7-d post-arrival to adapt to the LARU environment, and SYP + pens were fed phase 1 SYP + feed beginning on d-8 post-arrival. Two SYP + pigs were euthanized during phase 2 due to leg issues not associated with the treatment.

**Table 1:** Ingredients and composition of four-phase diets utilized in experiment #1 (as-fed basis).

	Phase^a^			
	1	2	3	4
**Ingredient, %**				
** Corn**	61.69	69.34	75.20	79.20
** Soybean meal, Dehull, Sol Extract**	33.90	26.55	20.95	17.05
** Soybean oil**	1.50	1.50	1.50	1.50
** Calcium carbonate**	0.85	0.85	0.85	0.85
** Calcium phosphate**	0.50	0.35	0.25	0.20
** Sodium chloride**	0.50	0.50	0.50	0.50
** L-lysine-HCl**	0.40	0.35	0.31	0.31
** DL-methionine**	0.15	0.09	0.04	0.05
** L-threonine**	0.02	0.02	0.02	0.02
** L-tryprophan**	0.02	0.02	0.02	0.02
** L-valine**	0.07	0.03		
** Trace mineral premix^b^**	0.15	0.15	0.13	0.10
** Vitamin premix without phytase^c^ **	0.15	0.15	0.13	0.10
** Quantum Blue 10G**	0.05	0.05	0.05	0.05
** Supplement^d^**	0.05	0.05	0.05	0.05
**Total**	100.00	100.00	100.00	100.00
**Calculated analysis**				
**Standarized ileal digestible (SID) amino acids, %**				
** Lysine**	1.32	1.10	0.93	0.79
** Isoleucine: lysine**	60	60	61	60
** Leucine: lysine**	121	129	139	143
** Methionine: lysine**	33	31	29	31
** Threonine: lysine**	61	61	61	64
** Tryptophan: lysine**	19.1	19.2	19.0	19.0
** Valine: lysine**	69	69	69	69
**Total lysine, %**	1.47	1.24	1.05	0.95
**Net energy, kcal/kg**	2496	2541	2576	2602
**Crude protein, %**	21.9	18.9	16.6	15.1
**Ca, %**	0.63	0.57	0.52	0.49
**P, %**	0.50	0.43	0.39	0.36

a Phase 1 d 0 to 21, phase 2 d 22 to 42, phase 3 d 43 to 63, phase 4 d 64 to 84. Both treatments were fed from 28.3 to 120 kg.

b Provided per kg of premix 73 g Zn from Zn sulfate; 73 g Fe from iron sulfate; 22 g Mn from manganese oxide; 11 g Cu from copper sulfate; 0.2 g I from calcium iodate; 0.2 g Se from sodium selenite.

c Provided per kg of premix: 3,527,399IU vitamin A; 881,850 IU vitamin D; 17,637 IU vitamin E; 1,764 mg vitamin K; 15.4 mg vitamin B12; 33,069 mg niacin; 11,023 mg pantothenic acid; 3,307 mg riboflavin.

d *Saccharomyces* yeast postbiotic (celluTEIN; Puretein Bioscience LLC, Minneapolis, MN) exchanged for corn at 0.45 kg per 907.2 kg total diet. Product contains dried yeast (97%), L-arginine (1.7%), L-leucine (1.3%), with a guaranteed analysis of 52% crude protein, 1% crude fat, 10% moisture, and 8% ash (minimums).

#### Feed performance

Every 7 d, pigs were individually weighed to calculate pen ADG. Additionally, pigs’ physical condition was observed and recorded. Unconsumed feed was vacuumed and weighed to calculate pen average daily feed intake (**ADFI**) and feed conversion ratio.

#### Harvest and carcass

On d-91, 98, and 105, the heaviest male and female pigs from each pen were transported to the University of Georgia Meat Science and Technology Center (Athens, GA) for harvest under federal inspection approved procedures. After 24-h postmortem chilling, the right carcass side was ribbed between the 10^th^ and 11^th^ ribs, loin eyes bloomed for 30 min, and carcass data were collected by trained university personnel. Back fat thickness was measured at the first, tenth, and last ribs by one trained personnel using back fat probe. Loin eye area was traced, scanned at 1200 × 1200 dpi resolution, and measured by using ImageJ (National Institutes of Health, Bathesda, MD). Loin eye color and marbling were subjectively evaluated by three trained university personnel and were averaged by using the National Pork Producers Council color and marbling scores (NPPC 1999) and Japanese loin and fat color scores (Nakai et al. 1975). Objective loin eye surface color values (Commision Internationale de l’Eclairage system [CIE, 1976], lightness [CIE L*], redness [CIE a*], and yellowness [CIE b*]) were measured using HunterLab MiniScan EZ (Hunter Associates Laboratory, Reston, VA) handheld spectrophotometer (Illuminant A, 10° observer, 2.54-cm aperture) at three locations and averaged.

### Experiment #2

Utilizing a different cohort of weaned pigs (60 barrows and 50 gilts; 25.9 ± 6.47 kg), all experimental procedures mimiced those employed during Experiment #1; however, diets were designed to contain a conventional protein amount (Table 2). During phase 2 feeding, pigs were affected by presence proliferative ileitis caused by *Lawsonia intracellularis*, which caused an increased removal rate. During phase 1, one SYP + pig was euthanized due to a leg issue unrelated to the treatment. During phase 2, one SYP- pig was euthanized due to a leg issue unrelated to the treatment, four SYP- and six SYP + pigs were removed due to ileitis. Two SYP + pigs were removed due to ileitis during phase 3, and one SYP- and one SYP + pigs were removed during phase 4 due to ileitis.

**Table 2:** Ingredients and composition of four-phase diets utilized in experiment #2 (as-fed basis).

	Phase^a^			
	1	2	3	4
**Ingredient, %**				
** Corn**	65.35	73.78	80.40	83.90
** Soybean meal, Dehull, Sol Extract**	30.70	22.45	15.95	12.55
** Soybean oil**	1.50	1.50	1.50	1.50
** Calcium carbonate**	0.60	0.60	0.60	0.60
** Calcium phosphate**	0.50	0.35	0.25	0.20
** Sodium chloride**	0.50	0.50	0.50	0.50
** L-lysine-HCl**	0.28	0.30	0.31	0.31
** DL-methionine**	0.08	0.04	0.02	0.02
** L-threonine**	0.08	0.07	0.08	0.09
** L-tryptophan**	0.01	0.01	0.03	0.03
** Trace mineral premix^b^**	0.15	0.15	0.13	0.10
** Vitamin premix without phytase^c^**	0.15	0.15	0.13	0.10
** Quantum Blue 10G**	0.05	0.05	0.05	0.05
** Supplement^d^**	0.05	0.05	0.05	0.05
**Total**	100.00	100.00	100.00	100.00
**Calculated analysis**				
**Standarized ileal digestible (SID) amino acids, %**				
** Lysine**	1.15	0.96	0.81	0.73
** Isoleucine: lysine**	64	62	60	59
** Leucine: lysine**	133	139	146	151
** Methionine: lysine**	31	30	28	30
** Threonine: lysine**	61	61	61	64
** Tryptophan: lysine**	19.6	18.7	19.0	19.0
** Valine: lysine**	69	69	69	69
**Total lysine, %**	1.29	1.08	0.91	0.83
**Net energy, kcal/kg**	2517	2570	2610	2634
**Crude protein, %**	20.4	17.2	14.6	13.3
**Ca, %**	0.54	0.46	0.42	0.38
**P, %**	0.49	0.42	0.38	0.35

a Phase 1 d 0 to 21, phase 2 d 22 to 42, phase 3 d 43 to 63, phase 4 d 64 to 84. Both treatments were fed from 18.7 to 110 kg.

b Provided per kg of premix 73 g Zn from Zn sulfate; 73 g Fe from iron sulfate; 22 g Mn from manganese oxide; 11 g Cu from copper sulfate; 0.2 g I from calcium iodate; 0.2 g Se from sodium selenite.

c Provided per kg of premix: 3,527,399IU vitamin A; 881,850 IU vitamin D; 17,637 IU vitamin E; 1,764 mg vitamin K; 15.4 mg vitamin B12; 33,069 mg niacin; 11,023 mg pantothenic acid; 3,307 mg riboflavin.

d *Saccharomyces* yeast postbiotic (celluTEIN; Puretein Bioscience LLC, Minneapolis, MN) exchanged for corn at 0.45 kg per 907.2 kg total diet. Product contains dried yeast (97%), L-arginine (1.7%), L-leucine (1.3%), with a guaranteed analysis of 52% crude protein, 1% crude fat, 10% moisture, and 8% ash (minimums).

#### Statistical analysis

Both experiments’ feed performance data were analyzed as completely randomized design with pen as the experimental unit. Treatment served as the fixed effect. Carcass data were analyzed as randomized complete block design with pen as the experimental unit. Treatment (**TRT**) served as the fixed effect and kill block served as random effect. All models were analyzed by using the MIXED procedure of SAS 9.3 (SAS Inst. Inc., Cary, NC). At *P ≤* 0.05 differences were considered significant and tendencies were considered at 0.05 > *P ≤* 0.10.

## Results 

### Experiment #1  

During the acclimation period (phase 0), there were no TRT effects for BW, ADG, and ADFI (*P >* 0.62; Table 3).  During phase 1, there were no TRT effects on BW, ADG, and ADFI (*P >* 0.14), but SYP + pigs had greater (*P <* 0.02) G: F ratio than SYP- pigs. During phase 2 and 3, there were no TRT effects for all measures (*P >* 0.12), except SYP- pigs’ phase 3 G: F ratio was greater (*P = *0.04) than SYP + pigs. During phase 4, there were no TRT effects on BW and ADG (*P >* 0.19), but SYP + pigs had smaller ADFI which resulted in greater G: F compared SYP- pigs (*P <* 0.04). Over all four phases, there were no TRT effects on ADG, ADFI (*P >* 0.13), except SYP + pigs had a greater (*P = *0.02) G: F ratio.   

**Table 3:** *Saccharomyces* yeast postbiotic effects on growth performance of pigs fed an elevated protein diet during four phases^a^.

	Treatment^b^			
	SYP-	SYP+	SEM	*P*-value
**Phase 0**				
** Body weight, kg**	28.3	28.4	0.34	0.80
** ADG^c^, kg/d**	0.99	1.01	0.028	0.62
** ADFI^d^, kg/pig d**	1.23	1.22	0.024	0.63
**Phase 1**				
** Body weight, kg**	49.4	50.4	0.64	0.28
** ADG, kg/d**	1.01	1.05	0.021	0.14
** ADFI, kg/pig d**	1.93	1.94	0.037	0.84
** G: F^e^**	0.54	0.56	0.006	0.02
**Phase 2**				
** Body weight, kg**	72.3	73.5	0.95	0.38
** ADG, kg/d**	1.08	1.09	0.023	0.86
** ADFI, kg/pig d**	2.42	2.44	0.035	0.74
** G: F**	0.45	0.45	0.010	0.86
**Phase 3**				
** Body weight, kg**	97.5	98.9	1.16	0.40
** ADG, kg/d**	1.20	1.21	0.016	0.70
** ADFI, kg/pig d**	3.15	3.26	0.049	0.12
** G: F**	0.39	0.37	0.004	0.04
**Phase 4**				
** Body weight, kg**	118.3	120.6	1.24	0.20
** ADG, kg/d**	0.99	1.04	0.023	0.19
** ADFI, kg/pig d**	3.51	3.31	0.064	0.04
** G: F**	0.28	0.31	0.007	<0.01
**All phases**				
** ADG, kg/d**	1.07	1.10	0.012	0.13
** ADFI, kg/pig d**	2.75	2.74	0.029	0.70
** G: F**	0.41	0.43	0.003	0.02

a Gilts (*N = *57) and barrows (*N = *51; PIC Camborough × 337 crosses) were weighed, stratified by body weight, and randomly assigned to 18 pens (*N = *6 pigs/pen) within each 18-pig strata.

b Dietary treatments consisted of 0 (SYP-) or 100 (SYP+) ppm saccharomyces yeast postbiotic (celluTEIN; Puretein Bioscience LLC, Minneapolis, MN) mixed into a high protein four-phase feeding regimen.

c Average daily gain; ADG.

d Average daily feed intake; ADFI.

e Gain to feed ratio; G: F.

#### Carcass characteristics  

There was no TRT effect on BW (*P = *0.40), but SYP + pig HCW tended to be heavier (*P = *0.09) than SYP- pigs which resulted in a greater (*P <* 0.01) dressing percentage (Table 4). There were no TRT effects on 10^th^ rib and last rib fat thickness (*P >* 0.13), but SYP + carcasses tended to have less (*P = *0.07) first rib fat thickness than SYP- carcasses. There were no TRT effects for loin area, NPB color score, Japanese fat color score, and 24-h L*a*b* values (*P >* 0.24). *Saccharomyces* yeast postbiotic carcasses had a smaller NPB marbling and Japanese marbling color scores than SYP- carcasses (*P <* 0.02).

**Table 4:** *Saccharomyces* yeast postbiotic effects on carcass characteristics of pigs fed an elevated protein diet during four phases^a^.

	Treatment^b^			
	SYP-	SYP+	SEM	*P*-value
**Body weight, kg**	132.4	133.6	3.23	0.40
**Hot carcass weight, kg**	103.1	105.4	2.64	0.09
**Dress, %**	77.8	78.9	0.25	<0.01
**Fat measures, cm**				
** First rib**	4.4	4.2	0.09	0.07
** 10^th^ rib**	2.7	2.6	0.18	0.19
** Last rib**	3.5	3.7	0.07	0.13
**Loin eye measures**				
** Area, cm^b^**	56.1	55.9	4.7	0.95
** NPB color score^c^**	2.3	2.4	0.14	0.55
** NPB marbling score^d^**	1.3	1.1	0.09	0.01
** Japanese fat color score^e^**	2.5	2.5	0.17	0.81
** Japanese marbling score^f^**	1.4	1.2	0.07	0.02
L*-value^g^	65.9	66.9	0.54	0.24
a*-value^h^	18.7	18.5	0.46	0.32
b*-value^i^	17.9	17.9	0.58	0.83

a Gilts (*N = *57) and barrows (*N = *51; PIC Camborough × 337 crosses) were weighed, stratified by body weight, and randomly assigned to 18 pens (*N = *6 pigs/pen) within each 18-pig strata. Pigs were harvested in three groups on days 91, 98, and 105 of feeding.

b Dietary treatments consisted of 0 (SYP-) or 100 (SYP+) ppm *Saccharomyces* yeast postbiotic (celluTEIN; Puretein Bioscience LLC, Minneapolis, MN) mixed into a high protein four-phase feeding regimen.

c National Pork Board (NPB) color scale based on a 6-point scale (1 = pale pinkish gray to white, 6 = dark purplish red; NPPC 1999).

d National Pork Board marbling scale based on 10-point scale (1 = 1% intramuscular fat content, 10 = 10% intramuscular fat content; NPPC 1999).

e Japanese pork color standard based on a 6-point scale (1 = pale grey, 6 = dark purple; Nakai et al. 1975).

f Japanese fat color standard based on a 4-point scale (1 = bright white, 4 = pinkish white; Nakai et al. 1975).

g Lightness; 0 = black and 100 = white.

h Redness; −60 = green and 60 = red.

i Blueness: 60 = Yellow; −60 = Blue.

### Experiment #2

During the acclimation period (phase 0), there was no TRT effect (*P = *0.97) on BW (Table 5). During phase 1 and phase 2, there were no TRT effects on all growth performance measures (*P >* 0.17). During phase 3, there was no TRT effect on ADFI (*P = *0.52), but SYP + pigs BW and ADG tended to be greater (*P = *0.08) and G: F was greater (*P <* 0.05) than SYP- pigs. During phase 4 and over all phases, there were no TRT effects on all growth performance measures (*P >* 0.12).

**Table 5:** *Saccharomyces* yeast postbiotic effects on growth performance of pigs affected by proliferative enteritis (*lawsonia spp*.) during a four-phase feeding trial^a^.

	Treatment^b^			
	SYP–	SYP+	SEM	*P*-value
**Phase 0**				
** Body weight, kg**	25.9	25.9	0.45	0.97
**Phase 1**				
** Body weight, kg**	46.1	47.6	1.11	0.33
** ADG^c^, kg/d**	0.97	1.03	0.035	0.21
** ADFI^d^, kg/pig d**	1.74	1.81	0.037	0.19
** G: F^e^**	0.55	0.58	0.016	0.21
** Removals**	0	1	—	—
**Phase 2**				
** Body weight, kg**	69.3	72.2	1.93	0.31
** ADG, kg/d**	1.07	1.06	0.042	0.87
** ADFI, kg/pig d**	2.39	2.33	0.075	0.59
** G: F**	0.44	0.46	0.013	0.40
** Removals**	5	6	—	—
**Phase 3**				
** Body weight, kg**	89.2	94.0	1.81	0.08
** ADG, kg/d**	1.06	1.13	0.029	0.08
** ADFI, kg/pig d**	2.92	2.99	0.073	0.52
** G: F**	0.35	0.38	0.009	0.05
** Removals**	0	2	—	—
**Phase 4**				
** Body weight, kg**	115.7	120.43	2.10	0.13
** ADG, kg/d**	1.16	1.15	0.028	0.74
** ADFI, kg/pig d**	3.26	3.22	0.097	0.79
** G: F**	0.35	0.35	0.009	0.63
** Removals**	1	1	—	—
**All phases**				
** ADG, kg/d**	1.06	1.09	0.025	0.42
** ADFI, kg/pig d**	2.58	2.59	0.052	0.88
** G: F**	0.42	0.44	0.009	0.12
** Total removals**	6	10	—	—

a Gilts (*N = *51) and barrows (*N = *57) were weighed, stratified by body weight, and randomly assigned to 18 pens (*N = *6 pigs/pen) within each 18-pig strata. Pigs were PIC Camborough × 337 crosses.

b Dietary treatments consisted of 0 (SYP-) or 100 (SYP+) ppm *Saccharomyces* yeast postbiotic (celluTEIN; Puretein Bioscience LLC, Minneapolis, MN) mixed into a conventional protein four-phase feeding regimen.

c Average daily gain; ADG.

d Average daily feed intake; ADFI.

e Gain to feed ratio; G: F.

## Discussion

### Experiment #1

Feed additives in the swine industry were used as diverse nutritional products to improve growth performance, health status, and carcass characteristics (DeRouchey et al. 2023). Price et al. (2017) concluded adding high-protein in growing-finishing pigs ­increased carcass dressing percentage when compared to ­commercial feed. Numerous studies demonstrated administering various yeast-based feed supplements had the potential to be an alternative to RAC because they enhanced BW, ADG, and feed efficiency (Shen et al. 2011; Dávila-Ramírez et al. 2020; Sun and Kim 2020). Vaughn et al. (2023) demonstrated SYP + played a role in regulating cell metabolism and muscle cell growth through the mTOR pathway. While SYP + has supplemental L-arginine and L-leucine, proprietary manufacturer information indicates when included at 100 ppm, less than 10 g of these amino acids are included per manufactured ton of feed. Therefore, all responses seen are due to the active ingredients of the product and not the animo acids. Laplante and Sabatini (2009) reported the mTOR pathway was stimulated by many ligands and this pathway was essential for skeletal muscle growth regulation. Kim and Duarte (2024) ­reported *Saccharomyces* yeast contained signaling ­molecules that activated target of rapamycin kinases. Therefore, exploring the effects of high-protein and SYP products on pig growth performance was the main direction of this study.

Dávila-Ramírez et al. (2020) reported 0.2% *Saccharomyces* yeast culture supplementation during growing-finishing increased ADG 5%, while 0.3% *Saccharomyces* yeast culture supplementation led to a 6% increase in ADG compared to control diets. Kim and Duarte (2024) demonstrated feeding 100 ppm SYP supplementation during nursery and finisher periods increased BW and ADG by 10 and 8%, respectively. Notably, these findings were generated using commercially designed nursey and finisher diets. In contrast to these studies, there were no differences in phase-specific and entire trail average BW and ADG during the current study. The difference in response between the current study and Kim and Duarte (2024) could be pigs were supplemented during the nursey phase which may have programmed them to have increased BW and ADG. In agreement with the current study, Li et al. (2025) reported pigs fed a Chinese-industry two-phase commercial growing diet supplemented with SYP had no change in phase 1 ADG, a tendency for a 0.1 kg increase in phase 2 ADG, which resulted in no SYP effect on the whole feeding trial’s ADG. These and the current trial may indicate timing of SYP supplementation may control its effect on growth.

While no positive SYP effects were seen on BW and ADG in the current study, supplementing SYP to grower and finisher diets did have an effect on several phases G: F. During phase 1, SYP + pigs had 0.02 smaller G: F, while they gave back that advantage during phase 3. During phase 4, SYP + pigs consumed 0.1 kg/pig d less feed which resulted in a 0.03 G: F improvement. These trends resulted in an 0.02 (5%) entire feeding trial G: F improvement. Kim and Duarte (2024) also reported supplementing growing-finisher pigs 100-ppm SYP increased G: F by 7%. During the grow-finish phase of their trial, Vaughn et al. (2023) reported feed conversion, the inverse of G: F, was smaller only for those pigs supplemented SYP during the nursey phase. This may indicate, SYP supplementation is needed earlier to maximize its effect during the grow-finish production stage. Additionally, the Kim and Duarte (2024) and Vaughn et al. (2023) studies were conducted feeding conventional diets, which when combined with the current study’s results, indicate a high-protein diet is not needed to maximize the SYP growth effect.

While SYP supplementation only elicited G: F improvements of pigs fed a high protein diet, it is important to consider the impacts on carcass quality. Numerous studies demonstrated mTOR ligands performed essential muscle growth control functions (for reviews see Wullschleger et al. 2006; Ilha et al. 2018). Yoshioka et al. (2024) reported yeast protein stimulated the mTOR pathway, thereby modulating myogenic regulatory factors, and Vaughn and Gonzalez (2022) the SYP utilized in the current study signals through the mTOR pathway. Additionally, the authors demonstrated SYP increased myotube diameter of differentiated myotubes *in-vitro* by promoting pig satellite cell differentiation and hypertrophy. Liao et al. (2022) reported feeding yeast protein increased myofiber diameter; therefore, one would expect SYP supplemented carcasses to be leaner.

In the current study, SYP + carcasses were 2% heavier HCW, 1.1% greater dressing percentage, had 0.2-cm less first rib back fat, and 0.2 smaller NPB marbling scores, which would indicate leaner carcasses. While there was no SYP effect for loin eye area, it could be hypothesized the greater HCW and dressing percentage could be due to heavier shoulder and Boston butt primal weights, but these data were not collected. Dávila-Ramírez et al. (2020) reported 0.2% SCE increased 5% HCW during growing-finishing phase. Similar to the current study, the authors did not find 0.2% SCE affected 10^th^ longissimus muscle area but they did report a four-point decrease in 24-h L*. Overall, carcass data from the current study demonstrated SYP + improved two important industry carcass measures without negatively affecting product color; however, the cost of the extra dietary protein to achieve these advantages needs to be considered by producers.

### Experiment #2

Upon determining an elevated-protein diet produced minimal advantages in growth performance which may not justify diet cost, SYP + effects when included in a commercial standard-protein diet were explored. Unfortunately, a proliferative enteritis (**PE**) outbreak during second study affected study pigs during phase 2 and presented as hemorrhagic dysentery symptoms which resulted in the removal of 10 pigs. The effects were seen throughout the study’s remainder with 14 pigs being removed due veterinarian recommendation, with nine pigs being SYP + pigs. While not an intended study factor, the finding SYP + pigs had 5 and 9% greater BW and G: F, respectively, during phase 3 could indicate SYP may have had the ability to reduce effects of a disease such as PE.

Healthy intestinal mucosal integrity is critical to optimal nutrient absorption, preventing heightened intestinal permeability (Kim and Duarte 2024). Proliferative enteritis diseases usually occur in the ileum, are caused by enterocyte overgrowth, and affected pigs’ ADG, ADFI, and G: F decline which can lead to various outcomes from slowed growth to sudden death (Guedes et al. 2002). Holyoake et al. (1994) reported non-hemorrphagic PE commonly occurred in pigs aged six to 24 weeks; therefore, feed additives that reduce negative PE effects can be valuable for navigating disease outbreaks such as PE.

Numerous studies demonstrated SYP supplementation had the potential to improve gut health, especially during challenge/stress situations. Duarte and Kim (2024) reported nursery pig SYP supplementation promoted crypt cell proliferation, increased immune response, which provided villi protection and reduced diarrhea incidence one week after weaning. Vaughn et al. (2025) reported SYP + improved growth performance of health challenged broilers reared on dirty litter. When examining a SYP that does not have the bioactive compounds present in SYP+, Hung et al. (2025) demonstrated SYP supplementation reduced nursery pig diarrhea incidence, but this was done without altering intestinal structure. Gormley et al. (2024) found SYP improved young pig fecal score when challenged with F18 + *Escherichia coli* by reducing Toll-like receptor 4 gene expression and increasing mammalian target of rapamycin gene expression. Therefore, while SYP + in the present study was unable to reduce removals, data indicated it may have helped improved growth performance during the heart of the PE outbreak; however, a controlled challenge study needs to be conducted to test this hypothesis.

## Conclusion

In conclusion, these studies demonstrate elevated dietary protein levels in combination with SYP + improved G: F and carcass measures of leanness, but the advantages may not justify the extra cost associated with the greater dietary protein content. This study also showed SYP + may have had potential to enhance gastrointestinal health and alleviate adverse effects associated with PE. Pigs supplemented with SYP + displayed better growth performance, less severe diarrhea, and improved feed efficiency after being affected by PE but these effects need to be tested in a controlled study.

## Disclosures

Dr. Mathew Vaughn is the Director of Research for Puretein Biosciences. He assisted in diet formulation and provided the celluTEIN used in the studies. He did not have input in data analyses but assisted in interpretation.

## LIST OF ABBREVIATIONS:

ADG: average daily gain
ADFI: average daily feed intake
BW: body weight
HCW: Hot carcass weight
LARU: Large Animal Research Unit
NPB: national pork board
PE: proliferative enteritis
RAC: ractopamine-hydrochloride
SYP: *Saccharomyces* yeast postbiotics
SYP-: 0 ppm Saccharomyces yeast postbiotic treatment
SYP+: 100 Saccharomyces yeast postbiotic treatment
SCE: *Saccharomyces* cerevisiae
TRT: treatment
